# Data governance for ethical usage of linked routine health data in South Africa: balancing privacy and data sharing.

**DOI:** 10.23889/ijpds.v9i5.2721

**Published:** 2024-09-18

**Authors:** Themba Mutemaringa, Andrew Boulle, Nicki Tiffin

**Affiliations:** 1 Computational Biology Division, Integrative Biomedical Sciences Department, University of Cape Town; 2 Centre for Infectious Disease Epidemiology Research, School of Public Health and Family Medicine, University of Cape Town; 3 The Provincial Health Data Centre, Health Intelligence Directorate, Western Cape Department of Health; 4 Wellcome Centre for Infectious Disease Research in Africa, Faculty of Health Sciences, University of Cape Town; 5 South African National Bioinformatics Institute, University of the Western Cape; 6 Computational Biology Division, Integrative Biomedical Sciences Department, University of Cape Town

The ability to collate data of healthcare clients from disparate health information systems in the public health system in the Western Cape province of South Africa presents opportunities for improved healthcare outcomes, but it also necessitates the development of systems to address risks related to the potential for data breach and privacy violations.

Here, we describe the data governance infrastructure for the Provincial Health Data Centre (PHDC) in the Western Cape Province of South Africa, which consolidates person-level health data from various sources to support healthcare management and research. We describe how PHDC data governance protects sensitive health data by ensuring compliance with privacy regulations and legislation, and demonstrate the adoption of key pillars of data governance in order to implement policies and procedures that safeguard patient privacy and promote responsible data use. We review downstream impacts of data collation including improved continuity of care, outbreak detection and research support, and how these may be facilitated within the data governance infrastructure.

The presentation highlights the need to balance the potential benefits of routine health data collation and secondary use with managing the risks to privacy and security for healthcare clients, and describes how this is addressed by the PHDC. Future areas to be considered include adoption of Trusted Research Environments (TRE) and the use of synthetic data, as well as identifying effective ways of enforcing data governance policies and monitoring compliance levels.

